# Effects of a lifestyle programme on accelerometer-measured physical activity level and sedentary time on overweight and obese women of Somali background living in Norway

**DOI:** 10.1186/s12889-025-22475-z

**Published:** 2025-04-07

**Authors:** Eivind Andersen, Linn Bohler, Maria J. Leirbakk, Danielle Cabral, Mia C. Wedegren, Mark L. Wieland, Haakon E. Meyer, Ahmed A. Madar

**Affiliations:** 1 Faculty of Humanities, Sports and Educational Science, University of South-Eastern Norway, Post box 2243, Tonsberg, N-3103 Norway; 2 Department of Community Medicine and Global Health, Institute of Health and Society, University of Oslo, Oslo, 0316 Norway; 3 Norwegian Institute of Public Health, Oslo, 0213 Norway; 4 District Sagene, Oslo Municipality, Vitaminveien 4, Oslo, 0485 Norway; 5 District Gamle Oslo, Oslo Municipality, Kolstadgata 1, Oslo, 0652 Norway; 6 Center for Health Equity and Community Engagement Research, Mayo Clinic, Rochester, MN 55902 USA; 7 Division of Community Internal Medicine, Geriatrics, and Palliative Care, Mayo Clinic, Rochester, MN 55902 USA

**Keywords:** Immigrant Somali women, Lifestyle programme, Overweight, Physical activity, Sedentary time, ActivPAL

## Abstract

**Background:**

Given the elevated prevalence and impact of overweight and the potential risk of non-communicable diseases among women of Somali background in high-income countries and recognising the potential positive impact of physical activity (PA) on these health conditions, it becomes imperative to focus on understanding the PA behaviour of this specific population. The objectives of this paper were twofold: firstly, to provide a comprehensive description of both objectively and subjectively measured PA level and sedentary time in a group of overweight women of Somali background in Norway, and secondly, to assess the effectiveness of a tailored, culturally sensitive, community-based intervention in increasing PA and reducing sedentary time.

**Methods:**

169 overweight women of Somali background in Norway were randomised by borough to either a lifestyle programme or a comparison group. The programme consisted of two sessions per week for 12 weeks, combining classroom discussion with graded group-based PA led by coaches, followed by monthly sessions over nine months. PA was measured objectively using the ActivPAL monitor and subjectively using the international PA questionnaire short form (IPAQ-SF) at baseline and 12 months after baseline.

**Results:**

The women took on average 6804 (SD = 3286) steps per day and were sedentary for 9.1 (SD = 3) hours per day at baseline. There were no differences between groups on any accelerometer measured PA variable at any timepoint. There were significant differences on vigorous intensity PA (25.9 min; 95% CI 7.7, 44.1) and total PA (77.6 min; 95% CI 13.2, 142.1) at the 12-month measurement session between the two groups using the IPAQ-SF.

**Conclusion:**

Despite observing initially low PA levels and high sedentary time at baseline and thus a considerable potential for intervention, the intervention failed in attaining an increase in accelerometer measured PA or reduction in sedentary time compared to the control condition. However, self-reported measures indicated success in these aspects. The potential threats to the programme’s reliability and validity include high drop-out rates possible due to the COVID-19 pandemic, contamination and low attendance rates. These challenges underscore the complexity of interventions in this demographic, emphasising the need for further exploration and refinement of methodologies to effectively enhance PA levels and reduce sedentary time in immigrant women living in high-income countries.

**Trial registration:**

clinicaltrials.gov NCT04578067, registered May 2021.

**Supplementary Information:**

The online version contains supplementary material available at 10.1186/s12889-025-22475-z.

## Introduction

The immigrant population constitutes 18.9% of the total Norwegian population of 5.4 M, with people of Somali descent representing 5.3% of immigrants [1]. Most Somali immigrants came to Norway in the early 2000s to escape conflict, civil unrest and instability in their home country [2]. A high prevalence of type 2 diabetes (T2D) has been found among women of Somali background in the U.S [3]., moreover, studies from Norway, Sweden, Italy, and New Zealand all found a prevalence of overweight/obesity above 50% (range 50.9–87.2%) [4–8]. In a study with 4194 immigrants from 12 different countries in Norway, women of Somali background had the highest odds of being overweight/obese [9]. Furthermore, the prevalence of overweight is higher among women with Somali background living in Norway compared to Somalis in Somali [8]. The length of residence in Norway, adjusted for age, is linked to a higher likelihood of developing T2D and overweight [10]. This association is likely a consequence of shifts in dietary patterns and physical activity (PA), driven by the processes of adaptation to the new country and urbanisation. These changes can be attributed to the influence of labour-saving technologies and the increased availability of high-calorie foods [11].

PA plays a pivotal role in the prevention and management of overweight and obesity [12], conditions strongly linked to the risk of T2D [13], cardiovascular diseases (CVDs) [14, 15], and overall mortality [16]. The impact of a lifestyle intervention on cardiovascular risk factors is tied to the degree of body weight loss [17]. Notably, the decrease in body fat is likely a major mediator in the relationship between changes in PA and alterations in risk factors for cardiometabolic diseases [18]. However, studies have shown that an increase in PA can affect biomarkers of cardiometabolic risk even when there is no concurrent reduction in body weight [19]. Furthermore, sedentary time - defined as any waking behaviour characterised by an energy expenditure ≤ 1.5 metabolic equivalents (METs) while in a seated, lying or reclining posture [20] - is found to be associated with higher mortality but may be somewhat mitigated by moderate-to-vigorous-intensity PA (MVPA) [21]. Research on the PA level of women of Somali background living in the global north is scarce, but consistent, in that all found the women to be little engaged in PA or exercise [3, 7, 22–24]. To our knowledge, data on sedentary time in this group has not been published. Although studies consistently show a low level of PA among women of Somali background in Norway and other high-income countries, the results may be flawed using self-reported methods when collecting PA data. These methods carry certain weaknesses. Immigrants of Somali background, and immigrants in general, may find it difficult to fill in questions that are not presented in their language, and validity evidence is lacking for PA assessment for Somali populations. In addition, accurate information about the duration and intensity of the activity is a problem. These are all methodological issues that can be overcome by using objective measurements such as an accelerometer. To our knowledge, there are no papers giving objectively measured PA levels and/or sedentary time in women of Somali background. Although accelerometers have been found to have good measurement properties to assess sedentary, standing, and stepping time and postural transitions in adults [25, 26], they place a bigger burden on the participants, are more expensive, time-consuming and require relatively higher technical expertise than self-report methods.

The Somali population appears to have shifted from a predominantly pastoral and nomadic way of life to a more settled one when emigrating to a high-income country, leading to a significant alteration in their daily PA routines [27]. In addition to labour-saving technologies and other advancements that enable reduced physical exertion, reasons for the low PA level may be lack of suitable places to exercise, cold weather and short daylight periods during winter, large family size and limited childcare resources, safety concerns, tradition and religious beliefs and norms [23, 24, 28, 29]. PA promotion is particularly crucial for groups at high risk of chronic diseases who experience disparities in PA participation. This includes immigrant women who face a plethora of barriers to PA. Despite calls for interventions to address PA among immigrant populations [30, 31], few interventions have been reported. To our knowledge, only three randomised controlled studies have been conducted with women of Somali background, where two were unsuccessful in increasing the participants’ PA level [32, 33] and one managed to increase the participants’ PA level, but had a small sample size (*N* = 27) and used self-reported measure of PA [34]. We lack knowledge of how best to promote awareness of the importance of PA and engage women of immigrant background in PA, but research suggests that community-based culturally safe approaches that are tailormade to the participants and accommodate culture, gender and socioeconomic factors are needed [35].

The study’s primary objective was to foster enduring changes in lifestyle through the application of motivational theories, specifically drawing from Self-Determination Theory [36] and Achievement Goal Theory [37]. The programme’s focus was to empower women in cultivating intrinsic and personally meaningful motivation to embrace a more active, less sedentary, and healthier dietary lifestyle. Notably, the programme was customised with a gender- and culturally sensitive approach, encompassing considerations in context, content, and delivery style and thus placed significant emphasis on adapting the intervention to align with the cultural norms, beliefs, and traditions of its participants.

Since the prevalence of obesity is especially high among women of Somali background in Norway and other high-income countries, and PA can beneficially influence this condition, special attention to the PA behaviour of this population group is needed. Based on the aforementioned limitations in existing research, the aims of this paper were firstly, to describe accelerometer measured, and subjectively measured PA patterns in a group of overweight and obese women of Somali background living in Norway, and secondly, to examine the long-term efficacy of the tailormade, culturally sensitive and community-based intervention in increasing PA and reducing sedentary time.

## Methods

### Study design

This study is based on data from the Increased Physical Activity and a Healthier Lifestyle among Immigrant Women study (ASLI) (clinicaltrials.gov NCT04578067, registered May 2021). The methods used in ASLI have been described in detail elsewhere [38]. Briefly, the study was designed as a randomised, controlled clinical trial to investigate the effect of a lifestyle programme (increased PA, reduced sedentary time (SED), and healthier eating) on weight loss. Thus, the current study is based on our secondary outcomes (PA and SED time). Results of the study on the primary variable (weight loss) gave modest non-significant effects on weight loss after 12 months [38]. To mitigate the potential risk of contamination between the intervention and comparison groups, treatment conditions were randomly allocated from two distinct boroughs. The study was approved by the Norwegian Centre for Research Data (ref: 724880). The comparison group received the programme after the 12-month measurement session.

### Recruitment and participants

Participants were recruited through Somali radio, women’s groups, general practitioners, and female user representatives from Somali community-based organisations who were included in the study team to help with information and recruitment, from September 2020 through May 2023 from two boroughs in Oslo, Norway. Women with a Somali background (born in Somali or parents born in Somali) aged 20 and above with a body mass index (BMI) of ≥ 27.0 kg.m^− 2^ were eligible for the study. Women who were pregnant, participating in a formal weight loss programme, taking medications that may affect weight loss, had severe musculoskeletal problems or difficulty walking, and had significant medical co-morbidities such as cancer, uncontrolled diabetes or diagnosed eating disorders, were excluded from the study. Written consent was obtained from all eligible participants who understood the nature of the research and were willing to participate.

### Intervention

The ASLI program was designed in collaboration with Somali women to support overweight and obese participants in achieving sustainable weight loss through increased physical activity (10 min or 1,000 steps daily), reduced sedentary time [30 min daily) [39], and healthier eating habits. The program also aimed to maintain these changes for at least 12 months post-baseline. Delivered at Healthy Life Centres in two Oslo boroughs, ASLI was implemented over 12 weeks for groups of 13–26 women, followed by monthly sessions for nine months. Each week included two 90-minute sessions combining classroom discussions with graded, group-based physical activity led by trained coaches.

ASLI fostered sustained lifestyle changes using motivational theories, including Self-Determination Theory and Achievement Goal Theory, to encourage internalized and self-relevant motivations for being more active, sitting less, and eating healthier. The program was gender-sensitive, ensuring its context, content, and delivery style aligned with participants’ cultural norms, beliefs, and traditions. Theory-driven mechanisms of behavior change (e.g., autonomous motivation, task-oriented goals) and evidence-based self-regulation techniques (e.g., self-monitoring, goal setting, implementation intentions) were central to the program. All materials, including informed consent forms, participant information, and food booklets, were translated into Somali, either in written or verbal formats.

The intervention was delivered by a multidisciplinary team of nutritionists, nurses, and public health specialists. Nutrition and physical activity (PA) manuals, developed by the research team, were based on recommendations from the Norwegian Directorate of Health. These manuals aimed to increase participants’ awareness of their PA levels, provide practical knowledge about PA, and guide strategies to overcome barriers, set goals, and prevent relapses.

ASLI coaches, who completed standardized two-day training, used a study-developed manual to provide participants with a “toolbox” of behavior change techniques. These techniques were reinforced through interactive, face-to-face group sessions. The materials and strategies were designed to help participants integrate new habits into their daily routines, ensuring long-term maintenance. Participants selected tools from the ASLI toolbox to modify their PA, sedentary behaviors, and diet, receiving simple, practical messages tailored to their individual needs and lifestyles.

The program emphasized fostering intrinsic motivation by helping participants recognize the personal value of the changes they made, such as increased energy and fitness. Peer support played a critical role, with participants collaborating to overcome challenges and encourage one another. The coaches fostered a positive, supportive atmosphere by emphasizing autonomy, respect for individual preferences, and mastery. They encouraged participants to set personally meaningful goals rather than imposing objectives, promoting sustained engagement through enjoyable, non-dogmatic, and interactive sessions.

The group dynamic was integral to ASLI’s success. Coaches created a team-like environment where women could share experiences, tips, and advice. Sessions emphasized mutual support, celebrating individual progress toward short-term goals, and recognizing efforts, not just achievements. Social support was extended beyond sessions through the use of social media platforms (e.g., Facebook, WhatsApp) and by encouraging participants to engage their broader social networks, such as family and friends, to reinforce changes.

Monthly follow-up sessions after the 12-week program provided participants with opportunities to share their experiences in maintaining lifestyle changes. These sessions reinforced the sense of community and offered continued motivation to sustain healthier behaviors. A more detailed description of the intervention is given in Additional file 2 (supplemental file 2).

### Measurements

Data collection took place at the Healthy Life Centres in the two boroughs. All measurements were conducted by a trained study team (nutritionist, nurse, epidemiologist, and a public health expert) who followed and used standardised protocols and tools. Each assessment included a physical examination and questionnaires, as previously described in detail [4].

#### Accelerometer measured PA

The two primary outcomes in this paper were total PA (10 min or 1000 steps per day) and total sedentary time (min per day), measured at baseline and 12 months after randomisation using the ActivPAL monitor (model ActivPAL micro; PAL Technologies, Glasgow, United Kingdom). The ActivPAL has been found to have good measurement properties to assess sitting, standing, stepping, and postural transitions in adults [25, 26, 40].

The ActivPAL is a compact activity monitor worn on the thigh for seven consecutive days. Researchers provided in-person instructions on how to properly attach the device during both visits and ensured that all participants successfully affixed it. Assistance was offered as needed to ensure participants could reapply the device themselves if it became detached during the monitoring period. Participants were instructed to wear the ActivPAL continuously, except during water-immersing activities such as swimming or bathing. After approximately seven days, participants returned to have the device removed, and the data were downloaded onto a computer for analysis. The data was analysed using PAL software suite version 8 and the enhanced analysis algorithm (CREA v1.3). Analysis was restricted to participants who wore the accelerometer for a minimum of 24 h per day (four-hour non-wear were allowed) for at least three days [41].

#### Subjectively assessed PA

The International PA Questionnaire Short Form (IPAQ-SF) [42] was used to assess subjective PA levels. The IPAQ-SF focuses on the amount of PA performed over the past seven-day period. The IPAQ-SF includes questions about the time spent engaging in vigorous PA, moderate PA and walking in 10-min bouts or longer. Within these domains, participants are asked to consider all types of physical activities, including activities performed during leisure time, domestic and gardening activities, work-related activities, and transport-related activities. Culturally relevant examples of activity performed at the different intensities were mentioned in the items. The questionnaire was in Norwegian, but if anyone had trouble understanding the question it was verbally translated into Somali language. The data obtained from the IPAQ-SF were used to estimate the total amount of PA completed in a seven-day period by weighting the reported min per week in each domain by a metabolic equivalent (MET) energy expenditure estimate. The weighted MET minutes per week were then calculated by multiplying the duration (minutes), frequency (days) and MET intensity and then summing the three domains, namely, vigorous (8 METs), moderate (4 METs) and walking (3.3 METs), to produce a weighted estimate of total PA per week (min∙week^− 1^) [43]. The participants were categorised into low, moderate and high PA levels according to the IPAQ scoring criteria [42]. Those categorised as having moderate or high activity were classified as being sufficiently active according to the WHO PA guidelines [44, 45]. Data cleaning and processing were carried out following the guidelines published by the IPAQ Research Committee, and the methods used to score the IPAQ are described in the IPAQ scoring protocol [42]. IPAQ has reasonable measurement properties for monitoring population levels of physical activity among adults in diverse settings [43].

#### Other measures

Weight was measured without shoes in light clothing by an Omron medical scale to the nearest 0.1 kg. Height was measured without shoes with a manual height measuring instrument (SECA stadiometer) to the nearest 0.1 cm. Waist circumference was measured midpoint between the lower margin of the last palpable rib and the top of the iliac crest using a measuring tape (SECA 201) to the nearest 0.1 cm with the participant standing and breathing normally. Overweight and obesity were defined as a BMI of 25–29 kg/m_2_ and ≥ 30 kg/m_2_, respectively. Data on education, employment, number of children in the household, years living in Norway, marital status, rating of programme and coaches, and whether or not they would recommend the programme to others were obtained through a self-complete questionnaire. Attendance was recorded by coaches.

### Statistical analysis

Statistical analyses were performed using SPSS (Statistical Package for the Social Sciences for Windows, version 29, IBM, Inc., Chicago, IL, USA), and multiple imputation analysis and linear regression after imputation was performed using STATA (version 17, StataCorp. LLC, Texas, USA). Descriptive data are presented as proportions, means, and standard deviations (SDs) with 95% confidence intervals (CIs) where appropriate. Within- and between-group differences in interval data were evaluated by t-tests (independent t-tests and paired t-tests). Correlations was used to analysis the associations between PA variables and attendance. For accelerometer measured and subjective PA analysis a multiple imputation with predictive mean matching was executed, assuming data is missing at random, and aiming to reduce bias and improve precision [46]. With this approach, the distribution of the observed data is used to estimate multiple possible values for the data points [46]. For multiple imputation and predictive mean matching, 40 imputations were used, reflecting the percentage of participants with a missing outcome, and ten was used as the number of nearest neighbours for predictive mean matching. A multiple imputation plot with an explanation is attached as additional file 1(supplemental file 1, Figures A1 and A2). Differences between the groups in terms of education level, employment and marital status were assessed using chi-squared tests. Associations with *p* < 0.05 (two-tailed) were considered significant.

## Results

**Fig. 1:**
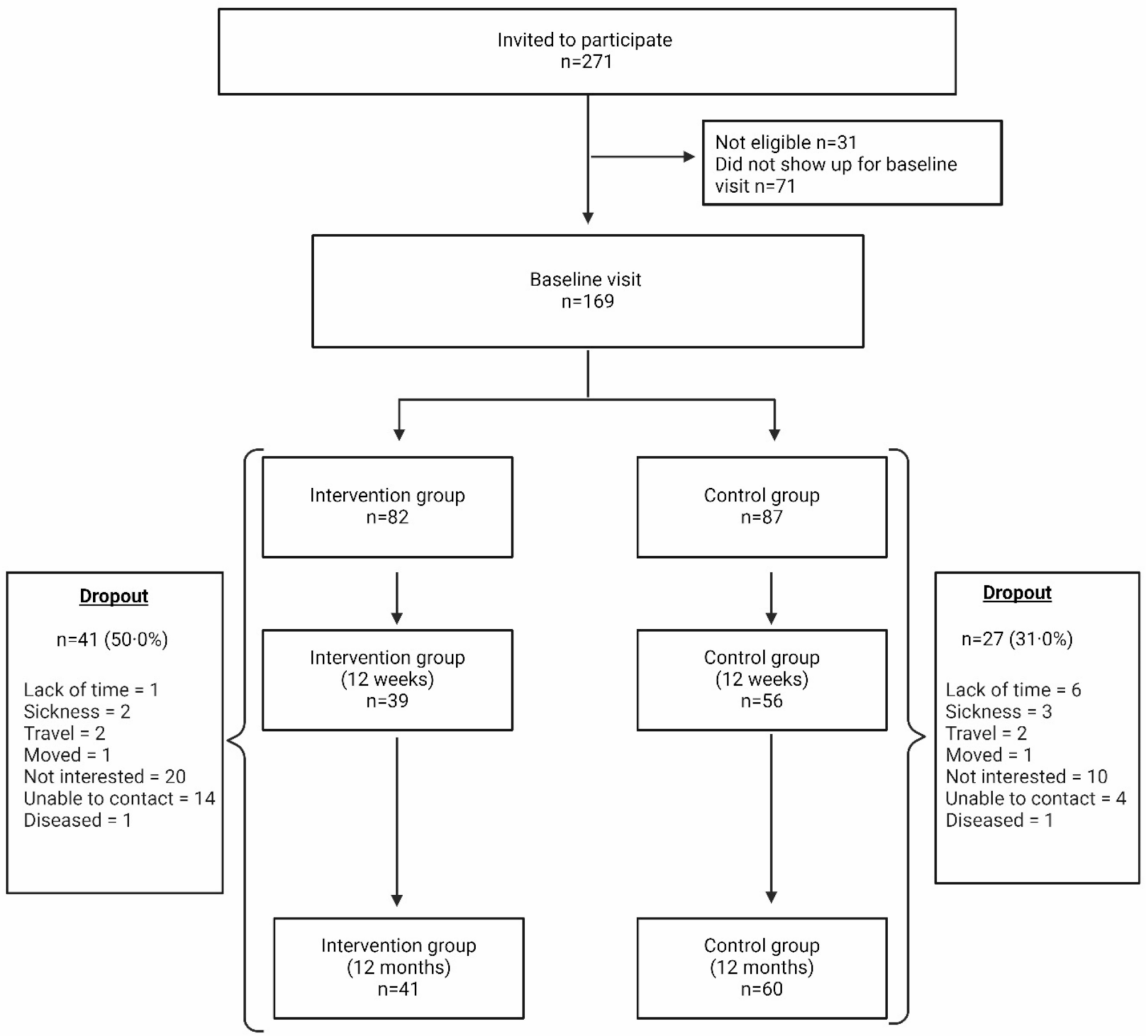
Flowchart showing follow-up of participants through the trial. *Adapted from Bohler et al. (2024) [38]

Of 271 women of Somali background invited to participate in the study, 169 accepted to participate, attended the first visit, and were found eligible. The reason for the exclusion of women who showed interest in the study was BMI < 27.0 kg.m^− 2^. Figure 1 shows the flow of participants through the trial. There was a significant difference between those with valid accelerometer recordings at the 12-month measurement session (*N* = 83) and those without valid accelerometer recordings (*N* = 86) on years of education of 1.4 years (95% CI = 2.7,0.1; *p* = 0.03). There were no other differences between those with valid accelerometer recordings at the 12-month measurement session and those without valid accelerometer recordings on any other background variables or PA level.

Table 1 shows relevant sociodemographic information for both groups. The women had lived in Norway for an average of 16 years, only one was born in Norway. Number of children in the household ranged from zero to seven. On average the women had completed around seven years of education ranging from zero to 16 years, where 7.8% had completed college or higher. Seventy-two per cent had a BMI above 30 kg.m^− 2^. The participants took on average 6804 (SD = 3286) steps per day and were sedentary for 9.1 h (SD = 3) per day at baseline. Twenty-eight (17 per cent) women reached 10,000 steps per day at baseline. There were no statistical differences between the groups at baseline.

**Table 1:** Baseline sociodemographic characteristics of participants allocated to the lifestyle programme immediately (intervention) or after 12 months (comparison). Data are mean (SD) or N (%)

Sociodemographic characteristics	Intervention (*n* = 82)	Comparison (*n* = 87)
Age (years)	45.8 (10.1)	47.9 (10.9)
Height (cm)	161.1 (4.9)	161.6 (4.5)
Weight (kg)	87.0 (12.9)	89.4 (14.5)
BMI (kg.m^− 2^)	33.5 (5.0)	34.1 (5.2)
Waist (cm)	99.1 (10.5)	97.2 (10.8)
Married (%)	43 (52)	61 (70)
Number of children living at home	2.2 (1.8)	2.2 (1.9)
Years living in Norway	16.3 (6.3)	16.8 (6.5)
Years in school	6.8 (4.4)	7.5 (4.2)
Working or studying (%)	42 (51)	43 (49)
PA (ActivPAL)		
Number of valid wear days	4.7 (1.8)	5.0 (1.9)
Steps per day	7175 (2975)	6455 (3536)
Sedentary time (min∙day^− 1^)	553 (174)	544 (187)
Stepping time (min∙day^− 1^)	96 (38)	85 (44)
Standing time (min∙day^− 1^)	258 (102)	252 (108)
Upright time (min∙day^− 1^)	354 (127)	337 (135)
PA (IPAQ)		
Walking (min.week^− 1^)	90 (90)	90 (60)
Moderate (min.week^− 1^)	0 (0)	0 (0)
Vigorous (min.week^− 1^)	0 (0)	0 (0)
Total PA (min.week^− 1^)	90 (90)	90 (90)

Abbreviations: SD, standard deviation; BMI, body mass index; PA, physical activity

Except for the number of valid days at the 12-month measurement, there were no differences between groups on any accelerometer measured PA variable regarding change from baseline to 12-month (Table 2). The number of steps per day and sedentary time were only 106 steps and 20 min higher in the intervention group compared to the control group with a very wide 95% confidence interval. Furthermore, there were no within-group differences in any of the PA variables between the baseline and post-programme time points (data not shown).

**Table 2:** Accelerometer measured physical activity and sedentary time outcome measures for participants in the intervention compared to a comparison group after multiple imputations (*n* = 169)

PA variable	Mean treatment difference (95%CI)^a^	Adjusted mean treatment difference (95%CI)^b^
ΔSteps per day	243.8 (-719.1, 1206.8)	106.1 (-870.3, 1082.5)
ΔSedentary time (min∙day^− 1^)	19.8 (-46.1, 85.7)	20.5 (-46.6, 87.5)
ΔStepping time (min∙day^− 1^)	6.5 (-5.9, 19.0)	4.8 (-7.9, 17.5)
ΔStanding time (min∙day^− 1^)	-9.5 (-54.2, 35.3)	-9.8 (-56.0, 36.5)
ΔUpright time (min∙day^− 1^)	-3.6 (-55.9, 48.7)	-5.3 (-59.5, 48.9)

Abbreviations: PA, physical activity; CI, confidence interval. ^a^Adjusted for baseline value and number of valid wear days at 12 months. ^b^Adjusted for baseline value, number of valid wear days at 12 months, age, education, employment, marital status, number of children in the household, and length of Norwegian residence

From a total of 24 sessions, the women attended 9.6 (6.5) sessions. 39% of the women attended more than half of the sessions and seven women did not attend any of the sessions. On average the women gave the programme a rating of 9.7 (0.5) on a scale of 1 to 10, and similarly rated the coaches at 9.7 (0.5). Furthermore, 95% would recommend the programme to others. There were no statistically significant correlations between change in steps or sedentary time with attendance recorded by the coach. There was no evidence that change in steps or sedentary time varied by age, years of education, number of children in the household, years living in Norway or baseline BMI.

Women achieving 10,000 steps per day at baseline, post-programme and 12 months in the intervention group was 15.8%, 11.9% and 15.8%, and for the comparison group, it was 20.3%, 12% and 12.2%. In the intervention group, 42.1% increased the number of steps from baseline to 12 months, compared to 48.9% in the comparison group, and 50% and 51.1% reduced sedentary time from baseline to 12 months, in the intervention and comparison groups, respectively.

There were no differences in self-reported PA levels between the two groups at baseline (data not shown). There were significant differences in change scores from baseline to 12-month for vigorous intensity PA and total between the two groups (Table 3).

**Table 3:** Subjectively assessed physical activity outcome measures for participants in the intervention compared to a comparison group after multiple imputations (*n* = 169)

PA variable	Mean treatment difference (95%CI)^a^	Adjusted mean treatment difference (95%CI)^b^
ΔWalking (min∙week^− 1^)	43.9 (-8.9, 96.6)	38.6 (-16.3, 93.5)
ΔModerate (min∙week^− 1^)	12.4 (-0.2, 25.1)	12.2 (-0.5, 24.9)
ΔVigorous (min∙week^− 1^)	26.6 (9.1, 44.1)^1^	25.9 (7.7, 44.1)^2^
ΔTotal PA (min∙week^− 1^)	82.5 (19.9, 145.1)^3^	77.6 (13.2, 142.1)^4^

Abbreviations: PA, physical activity; CI, confidence interval. ^a^Adjusted for baseline value, ^b^Adjusted for baseline value, age, education, employment, marital status, number of children in the household, and length of Norwegian residence. ^1^P-value = 0.004 ^2^P-value = 0.006, ^3^P-value = 0.010, ^4^P-value = 0.019

**Fig. 2:**
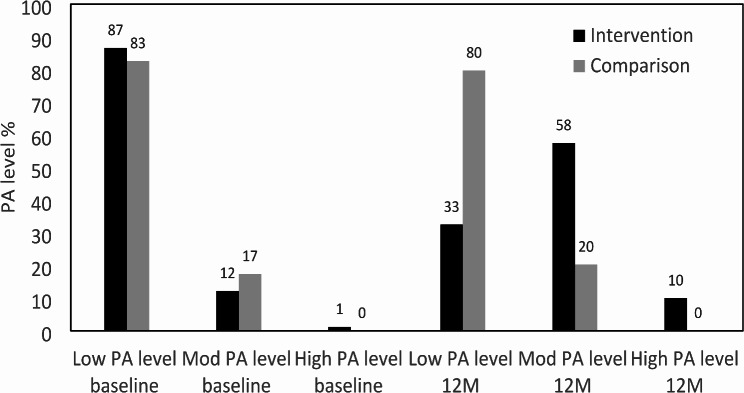
Distribution of PA level categories at baseline and 12 months, based on the IPAQ scoring protocol, for participants allocated to the lifestyle programme immediately (intervention) or after 12 months (comparison). Abbreviations: PA, physical activity; Mod, moderate; 12 M, 12 months

Most women in both groups were in the category of low PA level (not meeting the PA recommendations of 150 min of MVPA per week) at baseline (Fig. 2). A relatively large shift from the category low PA level to the categories moderate and high was seen for the intervention group, but not for the comparison group (Fig. 2). The chi-square test of independence showed that women in the intervention group were less likely to be in the low PA level category and more likely to be in the moderate and high PA level category at the 12-month measurement session than women in the comparison group (X^2^ (2, *N* = 99) = 23.9, *P* < 0.001).

## Discussion

This study aimed to determine both accelerometer-measured and subjectively measured PA level and sedentary time in overweight and obese women of Somali background in Norway. In addition, to examine the efficacy of the tailormade, culturally sensitive and community-based intervention in increasing PA and reducing sedentary time. We found that the women had a very low PA level and a high amount of sedentary time at baseline and that despite this high intervention potential, the intervention did not manage to increase accelerometer-measured PA level or reduce sedentary time more than the control condition, although about 50% of the women in the intervention group did increase PA level and reduced sedentary time. However, when measured subjectively, there was a highly significant difference in both vigorous intensity PA and total PA level between the groups at the 12-month measurement session.

The low accelerometer-measured PA level and high amount of sedentary time are in line with the findings of studies using self-reported measures in this group [3, 7, 22–24], and lower then a Norwegian population-based sample [47]. This is of big concern, seeing that PA level and/or sedentary behaviour are strongly linked to both morbidity and mortality [48, 49]. The reasons for these suboptimal behaviours are not known and are probably multiple and complex [23]. Although the study did take into consideration known barriers to PA in this group, and tailormade the intervention to their culture, moral norms, and beliefs we did not succeed in increasing PA levels or reducing sedentary time, even though they rated both the programme and the coaches very high and most of the women reported that they would recommend the programme to others. There may be several reasons for the lack of effect. First, the study was conducted during the coronavirus pandemic, and adherence to the intervention was difficult due to quarantine, being infected with COVID-19, and occasional lockdowns of schools and kindergartens. The programme’s lack of effect may thus be explained partly by the relatively poor attendance rate and that following the whole programme may have yielded different results, although we did not find a correlation between attendance rate and PA level. Second, another important reason for the lack of effect may be that many women focused on losing weight through dietary changes and not so much PA, and thus a stronger focus on PA and sedentary behaviour both in the programme and among the women may have provided different results. This is a very plausible explanation since weight loss, and not PA, was the main focus of both the study, and the women. Third, seeing that around half of the women in both groups increased PA levels and reduced sedentary time we cannot rule out the possibility of contamination between the boroughs due to being part of the same community across the city of Oslo [50]. Fourth, this study had a high dropout rate which made the study underpowered, and we cannot rule out a larger (or smaller) effect size if retention was higher. Fifth, the intervention season is a limitation, as it mainly took place during the autumn and winter months, due to Ramadan (the fasting month of Muslims), which is known to influence PA habits [51]. Lastly, although we do not have data on this, there could be that the women, despite voluntarily signing up for the programme, lacked motivation and commitment to change their PA level and sedentary behaviour, or that important aspects of the lessons and assignments were not fully grasped or understood.

An interesting observation emerged from our study, as there were no significant differences in the objectively measured PA levels between the two groups at the 12-month measurement session, but a noteworthy distinction did surface in the self-reported PA levels. This discrepancy could imply that the women genuinely perceive an increase in their PA level at the 12-month assessment compared to baseline. Alternatively, it raises the possibility of social-desirability bias, wherein participants may respond in a manner they believe aligns with societal expectations rather than providing accurate information. If the former holds true, with women feeling more physically active despite objective measurements suggesting otherwise, it may influence their attitudes and behaviours toward maintaining or further enhancing their PA levels. On the other hand, if social-desirability bias is at play, it prompts us to critically assess the reliability of self-reported data in capturing the true essence of participants’ experiences and behaviours in the study context.

The ASLI study has several strengths. First, a major strength of this research lies in its inclusive approach, wherein participants were not excluded based on their ability to speak or understand the Norwegian language thus increasing the generalisability of the study. This inclusivity was facilitated by the presence of a Somali-speaking researcher within the study team and the involvement of Somali user representatives in both boroughs. Second, based on previously reported barriers to PA in this group a major strength of the study is women-only groups, female trainers, information given in both Norwegian and Somali and tools and advice tailormade to Somali culture, norms, and beliefs. Third, a strength of the study is also the objective assessment of PA level and sedentary time, since self-reported measurements are known to have serious flaws. However, although a minimum of three days of measurement data is recommended, we cannot rule out the possibility that including only those with a full week of data would have yielded more reliable and valid data [41].

As discussed above, limitations to the study could be contamination, the relatively low attendance rate and the high drop-out from the study. Seeing that the Somali community is relatively small and that the women have several mutual meeting places we cannot rule out the possibility that contamination has occurred. It is highly likely that the participants from the intervention group have interacted with those from the comparison group and have shared details about the programme, which could have led to unintended changes in behaviour within the comparison group. This can compromise the study’s internal validity by confounding the true impact of the intervention. Furthermore, the relatively low attendance rates, mainly caused by the coronavirus pandemic, could have undermined the effectiveness of the intervention. If a significant number of participants do not regularly attend the programme sessions, it becomes challenging to assess the true impact of the intervention on PA levels. This limitation may also raise questions about the programme’s feasibility, acceptability, and relevance to the target population, affecting the generalisability of the study findings. Participant drop-out is a common challenge in these types of studies and can introduce bias, especially if the reasons for dropping out are related to the intervention or outcome measures. In response to an open-ended question, the primary reasons for dropping out were lack of interest, lack of time, and illness. When participants drop out due to dissatisfaction with the programme or other unforeseen factors, the study’s ability to draw accurate conclusions about the programme’s effectiveness diminishes. High drop-out rates may also affect the representativeness of the sample and introduce selection bias. Lastly, the power calculations were done on change in weight and not change in PA level, and thus the study may have been underpowered.

In summary, our study aimed to comprehensively assess the PA and sedentary behaviour of overweight and obese women with Somali background living in Norway, both objectively and subjectively, and evaluate the efficacy of a tailormade, culturally sensitive, and community-based intervention. Despite observing initially low PA levels and high sedentary time at baseline, the intervention did not yield a statistically significant increase in objectively measured PA or reduction in sedentary time compared to the control condition but managed to do so when using self-reported measures. Contamination, low attendance rate and high drop-out rate are possible major threats to the reliability and validity of the programme. These limitations underscore the complexity of conducting interventions in this demographic and emphasise the need for further exploration and refinement of study methodologies to increase PA levels and reduce sedentary time in immigrant women living in high-income countries.

## Electronic supplementary material

Below is the link to the electronic supplementary material.

Supplementary Material 1

Supplementary Material 2

Supplementary Material 3

## Data Availability

After publication, anonymised data underlying the results and analysis can be made available to researchers upon reasonable request to the corresponding author. A data access agreement needs to be signed in advance.

## Abbreviations

PA: Physical activity
IPAQ: International PA questionnaire
T2D: Type 2 diabetes
MET: Metabolic equivalent
CI: Confidence interval
ASLI: Increased Physical Activity and a Healthier Lifestyle among Immigrant Women study
BMI: Body mass index
SED: Sedentary
SD: Standard deviation
CVDs: Cardiovascular diseases
MVPA: Moderate- to vigorous intensity physical activity
