# Supplementary material for: Effects of a lifestyle programme on accelerometer-measured physical activity level and sedentary time on overweight and obese women of Somali background living in Norway
Source: BMC Public Health. 2025 Apr 7;25:1310. doi: 10.1186/s12889-025-22475-z (PMC11977904; doi:10.1186/s12889-025-22475-z)

**Additional file 1; multiple imputation plots**

The plot (Figure A1) shows the effect of the intervention on change in stepping time as a coefficient with the confidence interval from an adjusted regression. Line 1 shows the complete case with 82 observations. The next five lines show the effects in five randomly selected imputed sets from the 40 imputed sets of 169 observations. They show the variation between the imputed sets.

The last line shows the multiple imputation effect with 169 observations and 40 imputed sets. The effect here differs slightly from the complete case. The imputed sets vary quite a bit, but the confidence interval is narrower in all the randomly selected imputed sets. The multiple imputations for stepping time have removed skewness (bias) and have better precision and therefore the imputed dataset is more reliable.

**Figure A1. Forest plot of stepping time with a complete case of 82 observations (blue line), five randomly selected imputed sets and the multiple imputation effect with 169 observations (green line)**


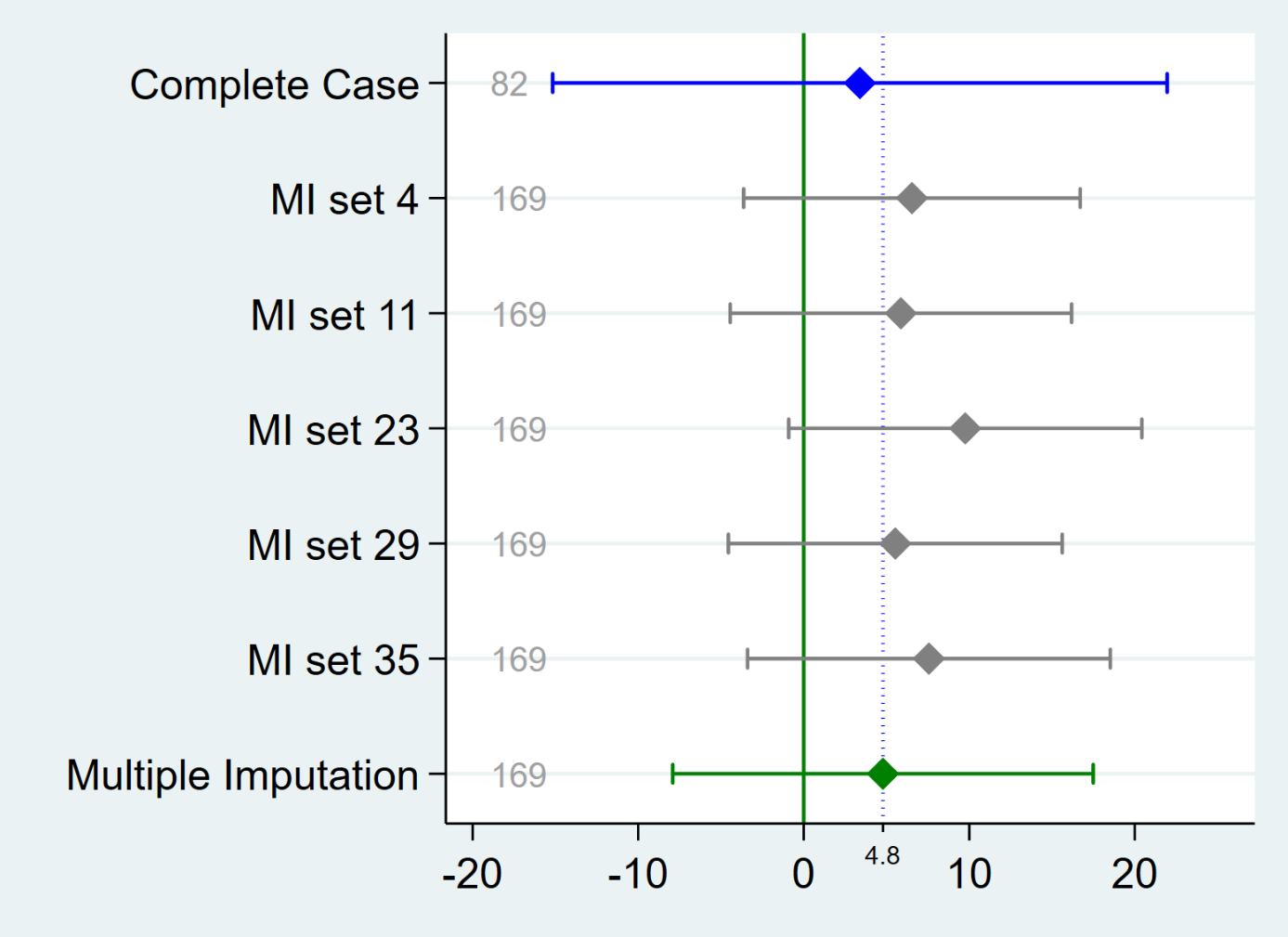


The plot (Figure A2) shows the effect of the intervention on change in sedentary time as a coefficient with the confidence interval from an adjusted regression. Line 1 shows the complete case with 82 observations. The next five lines show the effects in five randomly selected imputed sets from the 40 imputed sets of 169 observations. They show the variation between the imputed sets.

The last line shows the multiple imputation effect with 169 observations and 40 imputed sets. The effect here differs compared to the complete case, and since the imputed sets vary quite a bit, the uncertainty here is slightly greater. However, the precision is a bit better after multiple imputations.

**Figure A2. Forest plot of sedentary time with a complete case of 82 observations (blue line), five randomly selected imputed sets and the multiple imputation effect with 169 observations (green line)**


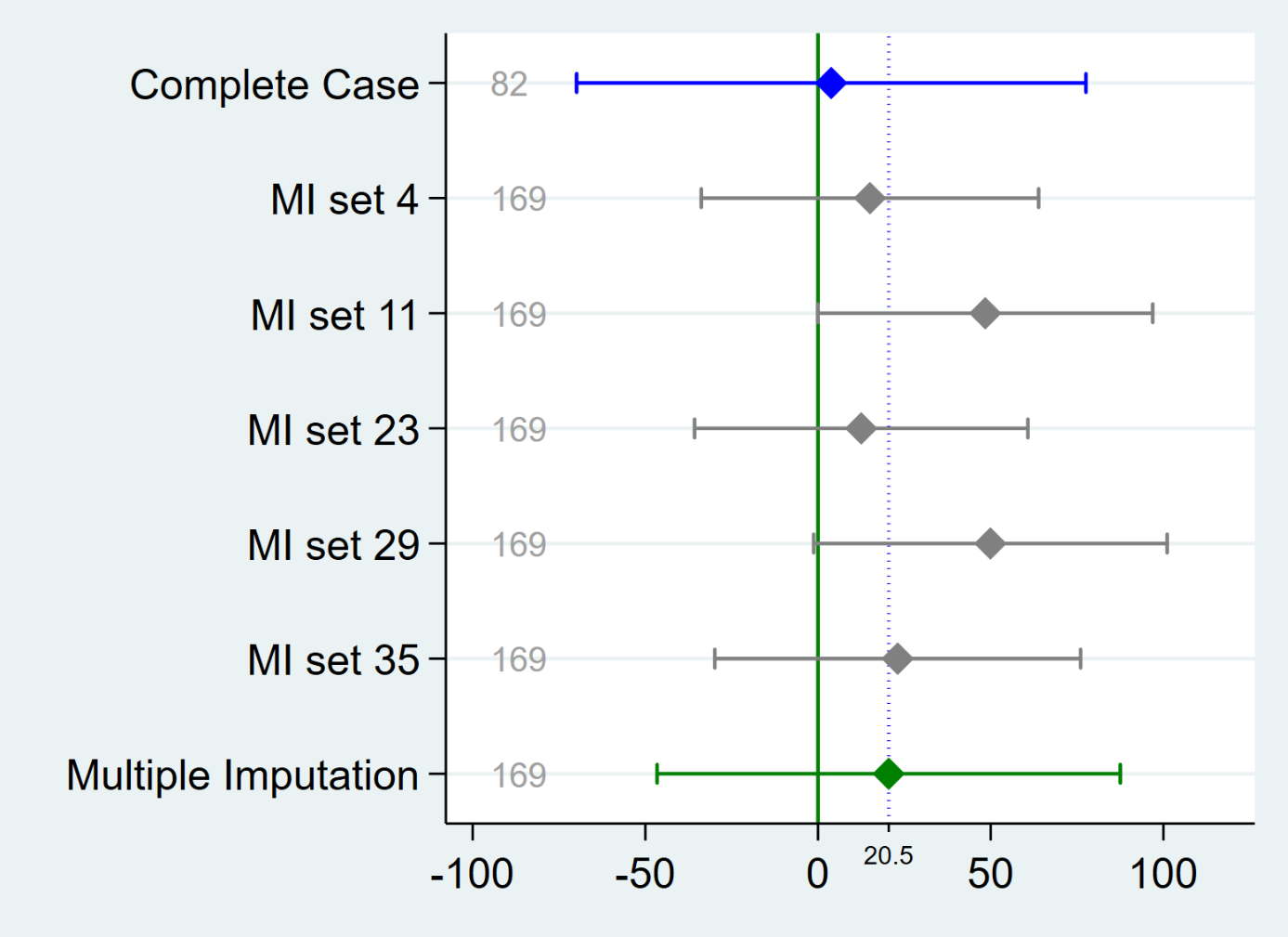

Supplement: Supplementary file 1 — Supplementary Material 1 [file 12889_2025_22475_MOESM1_ESM.docx]
