# Supplementary material for: Effects of a lifestyle programme on accelerometer-measured physical activity level and sedentary time on overweight and obese women of Somali background living in Norway
Source: BMC Public Health. 2025 Apr 7;25:1310. doi: 10.1186/s12889-025-22475-z (PMC11977904; doi:10.1186/s12889-025-22475-z)
Supplement: Supplementary file 2 — Supplementary Material 2 [file 12889_2025_22475_MOESM2_ESM.docx]

**Additional file 2: detailed description of the intervention**

Outline of group sessions in the intervention group

| **Week** | **Theme** | **Exercise** | **Behaviour change strategy** | **Targeted construct** |
| --- | --- | --- | --- | --- |
| 1 | Introduction: intention and expectations | Walking and light strength | -Improve knowledge and skills | -Self-efficacy  -Expectancies  -Environment |
|  | Goal setting: How do you set goals? | Balance and core muscles | -Improve goal setting for PA and food choice  -Increase social support | -Social support  -Self-efficacy  -Expectancies |
| 2 | Nutrition: Sugar and candy | Strength | -Improve nutrition and PA knowledge and skills  -Promote reduction in sugar sources | -Self-efficacy |
|  | How to manage and avoid pain | Yoga | -Improve knowledge on different forms of exercise: non-vigorous | -Self-efficacy |
| 3 | Barriers to exercise: psychological and physical benefits of exercise | Functional training | -Improve knowledge on how to incorporate PA into the daily routine  -Improve problem solving of PA barriers  -Promote positive outcomes of PA | -Social support  -Self-efficacy |
|  | Cardio: What happens when the pulse increases? | Pulse exercises: Speed/pace | -Improve knowledge on PA options, vigorous/non-vigorous  -Improve knowledge and skill to perform PA | -Self-efficacy  -Social support |
| 4 | Nutrition: Fat | Strength | -Improve nutrition knowledge  -Promote change of fat sources  -Improve knowledge and skills for PA | -Self-efficacy |
|  | Motivation: How to create motivation? What motivates? | Indoor cycling | -Enhance PA expectancies  -Improve goal setting for PA and food choice  -Promote positive outcomes of PA  -Improve knowledge on how to incorporate PA into the daily routine  -Improve knowledge and skill to perform PA | -Social support  -Self-efficacy  -Expectancies |
| 5 | Nutrition: Fruit and vegetables | Strength | -Improve nutrition knowledge  -Promote increase in intake of fruit and vegetables  -Improve confidence in strength exercise | -Self-efficacy |
|  | Why strength exercise? Why cardio exercise? | Pulse/cardio | -Improve knowledge on PA options, vigorous/non-vigorous  -Improve knowledge and skill to perform PA | -Self-efficacy |
| 6 | Nutrition: What does the food we eat contain and how to understand its content? | Yoga | -Improve nutrition knowledge and ability for healthier food choices  -Improve knowledge on different forms of exercise: non-vigorous | -Self-efficacy |
|  | Everyday functions: what can and should we think about? | Movement & Flexibility | -Improve knowledge on different forms of exercise: non-vigorous | -Social support  -Self-efficacy  -Environment |
| 7 | Nutrition: Portion control and what does the body need? | Balance | -Improve nutrition knowledge and ability for healthier food choices and smaller portions  -Improve knowledge on different forms of exercise: non-vigorous | -Social support  -Self-efficacy  -Environment |
|  | What is stress and how can we deal with it? | Strength/cardio | -Promote positive outcome of PA  -Improve knowledge and skill to perform PA | -Self-efficacy  -Social support  -Environment |
| 8 | Evaluation of previously set goals | Interval hill training | -Improve goal setting for PA  -Improve knowledge on how to incorporate PA into the daily routine  -Enhance PA expectancies | -Social support  -Self-efficacy  -Expectancies |
|  | Cooking: Bake bread fast and efficient | Participant’s choice | -Improve knowledge on healthy bread and easy baking  -Improve knowledge on different types of flour  -Improve social support  -Increase group dynamic  -Increase user involvement in PA | -Social support  -Self-efficacy |
| 9 | Nutrition: Somali compared to Norwegian food. What do we eat? | Participant’s choice | -Increase nutrition knowledge and raise awareness of food choices  -Increase group dynamic  -Increase user involvement in PA | -Social support  -Self-efficacy |
|  | What is obesity? And what does it do to the body? | Strength | -Increase health literacy  -Improve knowledge and skill to perform PA | -Self-efficacy |
| 10 | Nutrition: What is the keyhole label? | Balance with Bosu ball | -Improve nutrition knowledge and how to make healthier food choices  -Improve knowledge and skill to perform PA | -Self-efficacy |
|  | Good and bad habits and how they are created | Indoor cycling | -Enhance PA expectancies  -Improve goal setting for PA and food choice  -Promote positive outcomes of PA  -Improve knowledge on how to incorporate PA into the daily routine  -Improve knowledge and skill to perform PA | -Social support  -Expectancies  -Environment  -Self-efficacy |
| 11 | Nutrition: Is healthy food expensive? Cost-effective grocery shopping. | Strength | -Increase nutrition knowledge and knowledge on how to save money and sustain healthy eating  -Improve knowledge and skill to perform PA | -Self-efficacy |
|  | Family activities: What activities do we do with others? | Participant’s choice | -Increase social support for PA  -Provide opportunities for PA  -Improve knowledge on how to incorporate PA into the daily routine  -Improve knowledge and skill to perform PA | -Social support  -Self-efficacy  -Environment |
| 12 | Nutrition: What have we used from what we have learned? | Dynamic strength | -Increase nutrition knowledge  -Improve nutrition habits  -Improve knowledge and skill to perform PA | -Social support  -Self-efficacy |
|  | Sustaining change: How to enable lasting change | Speed and endurance | -Enhance PA expectancies  --Improve goal setting for PA and food choice  -Promote positive outcomes of PA  -Improve knowledge on how to incorporate PA into the daily routine  -Improve knowledge and skill to perform PA | -Social support  -Self-efficacy  -Expectancies |

Overview of the intervention components, behaviour change strategies and targeted social cognitive constructs

| **Intervention component** | **Dose** | **Description** | **Behaviour change strategy** | **Targeted construct** |
| --- | --- | --- | --- | --- |
| Group discussions | 45 minutes twice a week | The group discussions were held at the Healthy Life Centre prior to the structured group exercise. The project leader and the exercise instructor led the classes. Major topics were:  -Increased pulse  -Women’s health  -Setting small goals  -Motivation  -Identifying and reducing perceived barriers  -Seeking social support  -Home exercise  -Activity examples  -Healthy eating  -Key hole products  -Portion size  -Reading and understanding food labels  -Bread baking | -Improve health literacy  -Improve goal setting for PA and nutrition  -Improve problem solving for PA and nutrition barriers  -Improve social support for nutrition and PA  -Improve knowledge on how to incorporate PA into the daily routine  -Improve knowledge on PA options, both vigorous and non-vigorous  -Improve knowledge on healthy food choices | -Social support  -Expectancies  -Self-Efficacy |
| Structured group exercise | 45 minutes twice a week | The group exercise were held at the Healthy Life Centre exercise facilities. A female exercise instructor led the classes. The exercise-training programme was designed as a low threshold activity including yoga, balance with bosu ball, strength, cycling, core muscles and uphill walking, participant’s designing their own exercise session | -Promote self-confidence in exercising  -Promote user involvement  -Increase confidence in designing home exercise program  -Provide opportunities for exercise  -Improve knowledge and skills to exercise  -Increase social support  -Promote positive outcomes for an active life | -Self-efficacy  -Expectancies  -Environment |
