# Supplementary material for: Effects of a lifestyle programme on accelerometer-measured physical activity level and sedentary time on overweight and obese women of Somali background living in Norway
Source: BMC Public Health. 2025 Apr 7;25:1310. doi: 10.1186/s12889-025-22475-z (PMC11977904; doi:10.1186/s12889-025-22475-z)
Supplement: Supplementary file 3 — Supplementary Material 3 [file 12889_2025_22475_MOESM3_ESM.zip › Reviewer 1 Version 1 comments df9f3c42-e0c7-4087-a906-23222bb27669.docx]

Feedback for the author(s)

Comments to the authors

The manuscript, entitled "Effects of a lifestyle programme on objectively measured physical activity level and sedentary time on overweight and obese women of Somali background living in Norway" presents a study aiming to 1) describe physical activity (PA) level and sedentary time among overweight women with Somali background living in Norway and 2) investigate the effect of a culturally tailored lifestyle intervention program on the participants' physical activity level and sedentary time. The study had a randomized controlled design including 169 participants who were assessed at baseline and followed for 12 months. The intervention aimed to enhance physical activity and reduce sedentary time by incorporating motivational theories and considering cultural, gender, and socioeconomic factors. Physical activity was measured using both an objective device, the ActivPAL monitor, and a subjective tool, the International Physical Activity Questionnaire (IPAQ). The study found that the participants had an objectively measured PA level of mean 6804 (SD=3286) steps per day and 9.1 (3) hours of sedentary time. Furthermore, objective measures did not show a significant change in activity levels or sedentary time when compared to a control group, while self-reported physical activity increased more in the intervention group compared to the control group following the intervention.

This study has several strengths. It addresses a significant public health issue among a specific ethnic minority population with recognized health disparities, it employs a randomized controlled design to measure the effect of the culturally tailored intervention and uses an accelerometer (ActivPAL), which is considered more valid over self-report to assess PA level and sedentary time.

However, there are some ambiguities and issues throughout the manuscript which could be addressed to improve the manuscript. Please find my comments on specific sections in chronological order below.

Title

Your first aim is to describe the PA- and sedentary level among women with Somali backgrounds in Norway. Please consider including this aim in the title, in addition to the second aim which is to investigate the effect of the intervention.

Abstract

9.1 hours (SD=3) Please place the unit of sedentary time behind the (SD).

In the conclusion you write: "The potential threats to the programme's reliability and validity include contamination, low attendance rates, and high drop-out rates possible due to the COVID-19 pandemic. These challenges underscore the complexity of interventions in this demographic.." As it is written here, it seems like both contamination, low attendance rates, and high drop-out rates are possible due to COVID-19. And if that is true, the COVID-19-caused challenges were probably not demographic-specific and would be a challenge for intervention across many demographics at that time. Please revise to clarify.

Introduction

The manuscript addresses the prevalence of TD2 and overweight among immigrants of Somali background. It would have been interesting to know how this prevalence is compared to the population living in Somalia.

In the sentence "Research on the PA level of women of Somali background is scarce, but consistent, in that all found the women to be little engaged in PA or exercise", do you mean Somali women in Norway, immigrants with Somali background globally, or also women living in Somalia?

In the introduction you discuss the weaknesses of subjectively measured PA among previous studies to describe PA levels among women of Somali background in Norway (p.5) and to assess the effects of interventions (p. 6), however, you have chosen to use a subjective instrument to measure PA levels within the current study. I miss arguments for choosing this method in addition to the objective device. You argue that the accelerometers "..place a bigger burden on the participants, are more expensive, time-consuming, and require relatively higher technical expertise than self-report methods". (the argument has no reference). Despite these mentioned disadvantages, you have chosen to use an objective, in addition to a subjective, measurement method. A more throughout deliberation of the two devices’ strengths and weaknesses would probably help to understand your choices of methods. Could you describe the validity of ActiPAL compared to IPAQ to assess PA level and sedentary time?

Questionnaires are usually demographic/culturally sensitive. Is the IPAQ tool validated for this specific population?

The manuscript describes "..application of motivational theories, specifically drawing from Self-Determination Theory and Achievement Goal Theory". (p.6). A brief account of the most central components of the theories is needed – with references.

The arguments in this sentence (p.6) needs references: "Since the prevalence of obesity is especially high among women of Somali background in Norway and other high-income countries, and PA can beneficially influence this condition,.".

Methods

Study design

Please describe the treatment condition of the control group. Furthermore, in the Table legend in Table 1 it says that the comparison group was allocated to the lifestyle programme after 12 months. This information should be presented under Study design.

Recruitment and participants

The manuscript doesn’t describe any power calculations. Has there been conducted any power calculations? If not, this should be discussed as one of the study’s weaknesses.

One of the inclusion criteria was having a Somali background. How did you define/operationalize having a Somali background? (ref. in the results you mentioned that one of the participants was born in Norway).

Intervention

The manuscript has a detailed description of the intervention in terms of delivery techniques based on theoretical and cultural sensitivity. However, the specific content of the 90-minute session could have been more precisely described in terms of the type and dose of activity and themes in classroom discussions.

Measurements

Please specify what time points the measurements were assessed. It is somewhat confusing as the flowchart describes baseline, 2nd visit, and 3rd visit, whereas the text says baseline and 12 months after baseline.

"Each assessment included a physical examination, collection of blood samples, and questionnaires,.". (p.11). Outcomes assessed from blood samples are not included as part of the aim of this paper, please revise to clarify what measures that are relevant for the current study/paper.

Objectively assessed PA

The description of objectively measured PA lacks an explanation of how the data has been validated and processed, except for including only participants who wore the accelerometer for a minimum of 24 hours per day for at least three days. If there has been any programming of the device ahead of the assessment time, and/or any processing of the raw data to get the different outcomes, this should be reported to be able to repeat the measurements with the same procedures. I.e. are there any choices of resolution, thresholds for the different categories of activity, sedentary time, posture or similar?

For a day to be valid, did the participants have to wear the accelerometer for a minimum of 60 min per hour of 24 hours per day? What about exceptions such as water-immersing activities (e.g., swimming, bathing)?

Subjectively assessed PA

What language was the questionnaire in?

"Relevant examples of activity performed at the different intensities were mentioned in the items" (p.12). Were these examples culture-relevant?

"Those categorized as having moderate or high activity were classified as being sufficiently active according to the WHO PA guidelines (40)". Please note that these guidelines (WHO 2010) are outdated. WHO launched updated PA guidelines in 2020, where for instance the recommendation of 10-min bouts of PA is gone. This issue should be addressed somewhere in the manuscript.

The validity/measurement properties of the IPAQ tool within this specific population should be addressed.

Statistical measures

"The multiple imputation analysis and linear regression after imputation was performed using STATA" (p.13). In the first sentence under statistical measures, it says that "All statistical analyses were performed using SPSS" (p.13). Please revise.

Please specify in what analyses you have used linear regression.

What level of significance did you use?

Figure 1

Please explain when the 2nd and 3rd visits were, as you only have described two measurements; at baseline and 12 months after baseline in the manuscript.

Results

"Twenty-eight (17 per cent) women reached 10,000 steps per day at baseline" (p. 15). In the Intervention section (p. 8) it is described that "the intervention was designed to support the participants to lose weight through increased PA level (10 min per day), reduced sedentary time (30 min per day)" (p. 8). Furthermore, in the description of IPAQ; "The participants were categorized into low, moderate, and high PA levels according to the IPAQ scoring criteria (38). Those categorised as having moderate or high activity were classified as being sufficiently active according to the WHO PA guidelines (40)" (p. 12). Furthermore, the manuscript concludes that the participants had a low PA level and high sedentary level at baseline (the women had a very low PA level and a high amount of sedentary time at baseline (p. 19)), without comparing this to any other population or study results. To sum up, the manuscript lacks a unified operationalization of what the study has defined as being sufficiently physically active or as high/low PA level and a high/low amount of sedentary time. Please specify this somewhere in the manuscript.

"Except for the number of valid days at the 12-month measurement, there were no differences between groups on any PA variable at any time point" (p. 16). Please specify what measurement this is referring to.

"The number of steps per day and sedentary time were only 106 min and 20 min higher" (p. 16). Please revise 106 min to 106 steps.

On p. 17 you report results for attendance and rating of the program: "From a total of 24 sessions, the women attended 9.6 (6.5) sessions. Thirty-nine percent of the women attended more than half of the sessions and seven women did not attend any of the sessions. On average the women gave the programme a rating of 9.7 (0.5) on a scale of 1 to 10, and similarly rated the coaches at 9.7 (0.5). Furthermore, 95% would recommend the programme to others." (p.17)). The assessment of this information is not described in the methods. Please describe the methods of assessment of all data analysed and presented in this paper.

P. 18: "There were significant differences in vigorous intensity PA and total PA at the 12-month measurement session between the two groups (Table 3)". (p.18). Table 3 provides change- scores. Please report consistently whether you mean results at the 12-month assessment, or change from baseline to 12 months (treatment differences).

The study showed no differences between groups in objectively measured change in PA, however, there were significant differences between groups in subjectively measured change in self-reported PA. The study’s relevance could possibly be improved if you presented a cross-validation analysis between the two measurement methods.

Table 1

"Table 1 Baseline sociodemographic characteristics of participants allocated to the lifestyle programme immediately (intervention) or after 12 months (comparison)". (p. 16). The information about the comparison group being allocated to the lifestyle program after 12 months is not reported anywhere else in the manuscript. Please provide this information under the section Study design.

Figure 2

Please explain the abbreviations within Figure 2.

Discussion

As previously commented, please describe/operationalize what you define as a low PA level and high amount of sedentary time somewhere in the manuscript and consider comparing the study population’s level to the general population in Norway, or other comparable populations’ PA levels.

The manuscript discusses many relevant explanations of why the intervention did not show any effect on the objectively measured PA level and sedentary time, as well as strengths and weaknesses of the study. The manuscript mentions that "despite voluntarily signing up for the programme, lacked motivation and commitment to change their PA level and sedentary behaviour, or that important aspects of the lessons and assignments were not fully grasped or understood". (p. 20-21). The study intervention was designed with theoretical-based mechanisms to promote motivation for persistent PA. However, there is a lack of measures of how well the study achieved the theoretical goals mentioned in the methods (p.8), such as autonomous motivation, task-oriented goals, self-monitoring, goal setting, and implementation intentions. Could this issue possibly be addressed in the discussion?
