# Supplementary material for: Effects of a lifestyle programme on accelerometer-measured physical activity level and sedentary time on overweight and obese women of Somali background living in Norway
Source: BMC Public Health. 2025 Apr 7;25:1310. doi: 10.1186/s12889-025-22475-z (PMC11977904; doi:10.1186/s12889-025-22475-z)
Supplement: Supplementary file 3 — Supplementary Material 3 [file 12889_2025_22475_MOESM3_ESM.zip › Reviewer 1 Version 2 comments df9f3c42-e0c7-4087-a906-23222bb27669.docx]

Feedback for the author(s)

Thank you for the opportunity to review the revised manuscript. The authors have addressed all my comments, and either made clearifying adjustments or presented convincing arguments for not making suggested revisions. I find this revised manuscript, and the added supplemental file descripbing the intervention, clear and well written. I have no further comments.
