# Supplementary material for: Effects of a lifestyle programme on accelerometer-measured physical activity level and sedentary time on overweight and obese women of Somali background living in Norway
Source: BMC Public Health. 2025 Apr 7;25:1310. doi: 10.1186/s12889-025-22475-z (PMC11977904; doi:10.1186/s12889-025-22475-z)
Supplement: Supplementary file 3 — Supplementary Material 3 [file 12889_2025_22475_MOESM3_ESM.zip › Reviewer 2 Version 1 comments df9f3c42-e0c7-4087-a906-23222bb27669.docx]

Feedback for the author(s)

Thank you for the opportunity to review this study that describes device-based and subjectively assessed physical activity and sedentary time in a group of overweight women with Somali background in Norway, and secondly, assesses the effectiveness of a tailored, culturally sensitive, and community-based intervention in enhancing physical activity levels and reducing sedentary time.

The introduction gives a clear rationale for the study. The results have been presented appropriately and the discussion highlights the main findings of the study in relation to previous literature. However, there are several issues that could be described in more detail. These issues are specified in the following comments:

1. Abstract: Please indicate at which time points the measurements were conducted.

2. Abstract: Please provide some background characteristics of the participants, for example age, education etc.

3. Abstract: Please include some numerical results for the main findings.

4. Introduction/discussion: Please provide prevalence data of overweight/obesity and type 2 diabetes among Norwegian women to compare that with the prevalences among women with Somali background living in Norway. Also, population-based data on physical activity and sedentary time among Norwegian women would be informative to relate findings of the present study: How do 6804 steps and 9.1 hours of sedentary time at baseline relate to corresponding numbers among Norwegian women at the same age?

5. Introduction: I would suggest the use of for example the term device-based or accelerometer-measured instead of objectively measured since also device-based measurements include several subjective decisions on how the device is being used and how the data is being processed and analyzed.

6. Introduction: Please provide references for Self-Determination Theory and Achievement Goal Theory.

7. Methods, study design: Please write SED in full when used for the first time.

8. Methods, intervention: Please give more details and rationale for targeting at 10 min increase in PA and 30 min decrease in SED. Please give also details for healthier eating. Later in the methods it is stated that one of the primary outcomes was total PA (steps per day). How does this relate to minute-based increase in PA? Do you mean stepping minutes?

9. Methods, intervention: Please provide references for theory-derived mechanisms of action and for the most evidence-based self-regulation techniques.

10. Methods, intervention: Please specify the length of the intervention.

11. Methods, measurements: Please indicate the time windows for the measurements. Did you measure PA and sedentary time at 12 weeks?

12. Methods, Subjectively assessed PA: Please give details about the language version of the IPAQ-SF used. Was a validated translation used? It seems that both IPAQ and IPAQ-SF are used to refer to the same questionnaire. Please unify the acronym.

13. Methods, Statistical analysis: I would encourage you to present the results also without imputation of device-based PA. It can be expected that device-based PA is quite random, and imputation may affect the results as already shown in the supplementary file. It is obvious that when the n increases confidence intervals get narrower, and to my knowledge this is not an appropriate justification to favor imputation.

14. Results: How was the number of invited women (n=271) calculated from those reached by Somali radio, women’s groups, general practitioners, and female user representatives from Somali community-based organizations?

15. Results: There is a sentence “The number of steps per day and sedentary time were only 106 min and 20 min higher in the intervention group compared to the control group with a very wide 95% confidence interval.” Should there be “stepping time” instead of “number of steps per day”?

16. Table 1: The table title is confusing. According to manuscript text the table presents baseline characteristics of the participants in the intervention and control groups. Please specify.

17. Tables 2 and 3: It would be informative to see the results in both groups, not just the difference. And I would recommend you to report the results at baseline, at 12 weeks (if available) and at 12 months.

18. Results: It seems that the participants attended only less than half of the sessions. Did you conduct the analysis according to intention to treat or per protocol -principle?

19. Results: There is a sentence “There were no correlations between change in steps or sedentary time with attendance recorded by the coach.” However, correlation analysis was not described in the statical methods. And should there be “no statistically significant correlations” instead of just correlations?

20. Results: I suppose that instead of a chi-square test for example a regression analysis could be used to analyze differences between the groups so that also potential confounders could be controlled for.

21. Discussion: It is reported that “women focused on losing weight through dietary changes”. What kind of results were achieved and where have these been reported?

22. Discussion: Intervention season is mentioned as one of the limitations of the study. Why was the intervention conducted during the autumn and winter months while it was known beforehand that Ramadan influences PA habits of the participants? And I am a bit confused what you mean by intervention; the first 12 weeks or whole 12 months. You report the difference between baseline and 12 months but indicate autumn and winter months as an intervention season.

23. Discussion: Please add some discussion about the ability of the accelerometer used in this study to capture potential changes in PA and sedentary time.

24. Discussion: Related to my previous comments (11, 17 and 22) it seems that you measured PA at baseline and 12 months after the randomization. Why not after the 12 weeks of intervention? Please discuss potential effects of that.
